# Oxiracetam Offers Neuroprotection by Reducing Amyloid β-Induced Microglial Activation and Inflammation in Alzheimer's Disease

**DOI:** 10.3389/fneur.2020.00623

**Published:** 2020-07-17

**Authors:** Heng Zhang, Longfei Jia, Jianping Jia

**Affiliations:** ^1^Innovation Center for Neurological Disorders and Department of Neurology, Xuanwu Hospital, Capital Medical University, Beijing, China; ^2^Beijing Key Laboratory of Geriatric Cognitive Disorders, Beijing, China; ^3^Clinical Center for Neurodegenerative Disease and Memory Impairment, Capital Medical University, Beijing, China; ^4^Center of Alzheimer's Disease, Beijing Institute for Brain Disorders, Beijing, China

**Keywords:** Alzheimer's disease, microglia, inflammation, oxiracetam, neuronal protection

## Abstract

**Background:** Alzheimer's disease (AD) is characterized by amyloid beta (Aβ) accumulation in the brain, which triggers the activation of microglia; in turn, microglia release neuroinflammatory factors capable of damaging neurons. Thus, a therapeutic approach targeting this sustained microglia-induced inflammatory response deserves investigation. Here, we examined whether oxiracetam (ORC), a nootropic of the racetam family, can indirectly prevent Aβ-induced neurotoxicity by attenuating microglial activation.

**Methods:** Aβ42 oligomers were used to stimulate BV2 microglial cells, and the morphological changes and phagocytic capacity of BV2 cells were evaluated using fluorescence microscopy. We used quantitative reverse transcription polymerase chain reaction to assess the inhibitory effects of ORC on Aβ-induced mRNA levels of interleukin-1β (IL-1β), IL-6, and tumor necrosis factor-α (TNF-α); enzyme-linked immunosorbent assay was used to examine the productions of these cytokines. We also assessed the mRNA level of inducible nitric oxide synthase and the production of nitric oxide (NO). The conditioned medium from BV2 cells was used to culture hippocampal HT22 cells to assess indirect toxicity using the MTT assay.

**Results:** ORC prevented the Aβ-induced activation of BV2 cells, as reflected by reduced morphological changes and phagocytic ability. In addition, ORC downregulated the expression of Aβ-induced cytokines (IL-1β, IL-6, and TNF-α) and the production of NO in BV2 cells. Furthermore, ORC protected HT22 cells from indirect damage evoked by Aβ-treated BV2 cell-conditioned medium, but not from direct Aβ-induced toxicity.

**Conclusions:** ORC suppressed the activation of BV2 cells, decreased the production of Aβ-induced inflammatory molecules and NO in BV2 cells, and protected HT22 cells against indirect toxicity mediated by Aβ-treated BV2 cell-conditioned medium. Thus, ORC may exert a protective role in AD through attenuating the damage caused by inflammation and oxidative stress.

## Introduction

Alzheimer's disease (AD) is a chronic, progressive neurological disorder associated with a decline of cognitive function (1). Histopathologically, the brain of the patients with AD has two hallmarks: the extracellular accumulation of amyloid-β (Aβ) to form senile plaques and the intracellular hyperphosphorylation of tau to form neurofibrillary tangles (2). Aβ peptides, particularly Aβ oligomers, play a primary role in the pathogenesis of AD (3). Aβ oligomers are considered the most toxic form of Aβ peptides; they can trigger neuroinflammation (4), cause neuronal death (5), and impair synaptic plasticity (6).

Multiple studies suggest that there is a sustained inflammatory response, oxidative stress, and activated microglial clustering around Aβ accumulations in the brain of patients with AD (7, 8). Evidence has emerged to suggest that this sustained inflammatory response is another core feature of AD and that microglia are important mediators of Aβ-induced neuroinflammation and oxidative stress. When stimulated with Aβ, microglia are activated and release pro-inflammatory and neurotoxic factors such as interleukin-1β (IL-1β), IL-6 tumor necrosis factor-α (TNF-α), and nitric oxide (NO). In turn, these factors promote neuronal degeneration, ultimately inducing reactive microgliosis (9–11). Other evidence supports the idea that activated microglia can directly damage neurons (e.g., microglia can mediate the loss of synapses by engulfing synaptic components via the complement system) (12); they can also exacerbate the phosphorylation, aggregation, and spread of misfolded tau (13, 14). Thus, preventing Aβ-induced microglial activation, neuroinflammation, and oxidative stress may be a promising therapeutic strategy to improve the symptoms of AD pathology.

Oxiracetam (ORC) is a nootropic of the racetam family; it has been examined for its potential use in the treatment of cognitive impairment (15), cerebrovascular diseases (16), and multi-infarct dementia (17), because it can readily pass through the blood–brain barrier (BBB) and act selectively on the cortex and hippocampus (18). Recent reports have suggested that ORC improves memory in a rat model of vascular dementia and promotes recovery of cognitive function in a rat model of cerebral hypoperfusion (19). Furthermore, ORC remarkably reverses cognitive decline in older human subjects (20). Another study has suggested that ORC reduces the release of inflammatory cytokines in a rat model of stroke (21). However, whether ORC improves cognitive decline by preventing Aβ-induced inflammation and oxidative stress in AD models remains unknown; moreover, the mechanisms underlying its effects should be explored in more detail.

Here, we aimed to investigate whether ORC can prevent Aβ-induced microglial activation, inflammation, oxidative stress, and protect against Aβ neurotoxicity.

## Materials and Methods

### Materials

Dulbecco's modified Eagle's medium (DMEM) and fetal bovine serum (FBS) were purchased from Gibco (Grand Island, NY, USA). ORC was supplied by Shijiazhuang Pharmaceutical Group Ouyi Pharma Co., Ltd. (Shijiazhuang, China). Enzyme-linked immunosorbent assay (ELISA) kits were supplied by Cusabio Biotech (Wuhan, China). The NO assay kit was obtained from Jiancheng Bioengineering Institute (Nanjing, China). Dimethyl sulfoxide (DMSO), MTT, and latex beads were supplied by Sigma-Aldrich (Saint Louis, Missouri, USA). Aβ42 oligomer powder was obtained from ChinaPeptides Co., Ltd. (Suzhou, China). The RNA extraction kit was obtained from Sangon Biotech (Shanghai, China). TB Green® Premix Ex Taq™ II and PrimeScript™ RT reagent kits were obtained from Takara (Beijing, China). ActinRed was purchased from KeyGEN BioTECH (Nanjing, China).

### Preparation of Aβ42 Oligomer Solution

Briefly, 1 mg of Aβ42 oligomer powder was dissolved in DMSO to obtain 1 mM of stock solution, which was further diluted with DMEM to a final concentration of 5 μM. The soluble fraction was stored at −80°C.

### Cell Culture and Treatments

Murine microglial cells (BV2) were supplied by the National Infrastructure of Cell Line Resource (Beijing, China). The hippocampal neuronal cell line HT22 was obtained from LMAI Bio (Shanghai, China). Cells were maintained in DMEM containing 10% FBS at 37°C and 5% CO_2_.

All experimental procedures involving BV2 cells were conducted after overnight seeding and subsequent serum starvation for 0.5 h. For the phagocytosis assay and for assessing changes in microglial morphology, cells were cultured in 24-well plates (2.5 × 10^4^ cells/well). For analysis of pro-inflammatory cytokine and inducible nitric oxide synthase (iNOS) mRNA levels, cells were cultured in six-well plates (2 × 10^5^ cells/well). Cells were pretreated with ORC for 2 h after serum starvation and then co-cultured with Aβ for 10 h. For the analysis of pro-inflammatory cytokine and NO production, cells were pretreated with ORC for 2 h after serum starvation and then co-cultured with Aβ for 22 h. For the experiments mentioned above, four groups were classified as follows: (1) control BV2 cells; (2) Aβ-stimulated BV2 cells; (3) Aβ-stimulated BV2 cells treated with ORC; and (4) control BV2 cells treated with ORC.

For examination of indirect toxicity, conditioned media were obtained from three groups: (1) control BV2 cells; (2) Aβ-stimulated BV2 cells; and (3) Aβ-stimulated BV2 cells treated with ORC. BV2 cells in six-well plates were pretreated with ORC for 2 h and then co-cultured with Aβ for 10 h. After intervention, the medium was replaced with fresh medium without Aβ or ORC; 12 h later, the supernatant was collected as the conditioned medium.

For the MTT assay, 3 × 10^3^ HT22 cells were cultured in 96-well plates and then treated with conditioned medium for 24 h. For the direct toxicity experiments, HT22 cells were treated with different concentrations (5, 10, 20, and 100 μM) of ORC for 2 h after serum starvation for 2 h and finally co-cultured with Aβ for another 22 h.

### MTT Assay

Cell viability was examined using the MTT assay. After relevant incubations, cells were exposed to MTT for 4 h, after which DMSO (150 μl per well) was added, and optical density at 570 nm was recorded. The data are represented as a percentage of the viability in the control groups.

### Microglial Phagocytosis Assay and Morphological Characterization

The procedures for the microglial phagocytosis assay were referenced from the study by Lian et al. (22). Latex beads were pre-incubated in FBS for 1 h at 37°C at a ratio of 1:5 before dilution to a final concentration of 0.01% (v/v) and 0.05% (v/v) in DMEM. After BV2 cells were treated with Aβ with/without ORC, the BV2 cell culture medium was replaced by bead-containing DMEM, and cells were incubated at 37°C for 1 h. Thereafter, cells were washed and fixed in 4% paraformaldehyde for 15 min. The cytoskeleton was then probed using ActinRed (1:50) to obtain composite images that would allow the counting of phagocytic cells. The number of phagocytic cells/the total cell number was calculated to show the scale of activation of BV2 cells. Phagocytic efficiency, which was calculated based on a weighted average of engulfed beads per cell, was also determined to evaluate phagocytic ability as previously described (23): phagocytic efficiency (%) = (1 × *X*_1_ + 2 × *X*_2_ + 3 × *X*_3_ + 4 × *X*_4_ + 5 × *X*_5_ + 6 × *X*_6_)/(total number of cells) × 100%, where *X*_*n*_ represents the number of cells containing *n* beads. We chose distinct single BV2 cells to analyze the relevant morphological characteristics. The parameters quantified were as follows: morphology, area, perimeter, and Feret's diameter using Image J (Version 1.53a).

### Quantitative Reverse Transcription Polymerase Chain Reaction

Quantitative reverse transcription polymerase chain reaction (qRT-PCR) was used to evaluate levels of IL-1β, IL-6, IL-10, TNF-α, and iNOS mRNA. Total BV2 cell RNA was extracted and then reverse-transcribed into cDNA using the PrimeScript™ RT Reagent Kit. Quantitative PCR was performed using TB Green® Premix Ex Taq™ II, as follows: pre-incubation at 95°C for 30 s followed by 40 cycles of denaturation at 95°C for 5 s and annealing at 60°C for 30 s. The oligonucleotide primer sequences are shown in Table 1.

**Table 1:** Primers used for quantitative reverse transcription polymerase chain reaction.

**Name**	**Sequence**
IL-1β forward primer	5′-TTTCCTCCTTGCCTCTGATGGG-3′
IL-1β reverse primer	5′-CCACACGTTGACAGCTAGGTTC-3′
IL-6 forward primer	5′-CTTGGGACTGATGCTGGTGACA-3′
IL-6 reverse primer	5′-GCCTCCGACTTGTGAAGTGGTA-3′
TNF-α forward primer	5′-GTGGTCAGGTTGCCTCTGTCTC-3′
TNF-α reverse primer	5′-TGGCTCTGTGAGGAAGGCTGTG-3′
iNOS forward primer	5′-GGACGAGACGGATAGGCAGAGA-3′
iNOS reverse primer	5′-TCTTCAAGCACCTCCAGGAACG-3′
IL-10 forward primer	5′-CCCAGAAATCAAGGAGCATT-3′
IL-10 reverse primer	5′-TCACTCTTCACCTGCTCCAC-3′
GAPDH forward primer	5′-GAAGGGCATCTTGGGCTACAC-3′
GAPDH reverse primer	5′-GTTGTCATTGAGAGCAATGCCA-3′

*IL-1β, interleukin-1β; IL-6, interleukin-6; TNF-α, tumor necrosis factor-α; iNOS, inducible nitric oxide synthase; IL-10, interleukin-10; GADPH, glyceraldehyde 3-phosphate dehydrogenase*.

### ELISA Analysis

ELISA kits were used to evaluate the effects of ORC on the levels of IL-1β, IL-6, and TNF-α. In brief, after the corresponding treatments, the levels of pro-inflammatory factors in the cell supernatant were measured following the manufacturer's instructions.

### Nitric Oxide Assay

The concentrations of NO in culture supernatants were examined by measuring nitrate and nitrite, the major products of NO, following the manufacturer's instructions of the NO assay kit. The optical density was assessed at 550 nm.

### Statistical Analysis

For all group comparisons, one-way analysis of variance followed by Tukey's *post-hoc* test was performed. GraphPad Prism V8.0 (GraphPad Software Inc., California, USA) was used to analyze the data and images. All results are represented as the mean ± standard deviation. *p* < 0.05 was considered to be statistically significant.

## Results

### Oxiracetam Is Not Toxic to BV2 or HT22 Cells, Even at the Maximum Concentrations Tested

**Figure 1:**
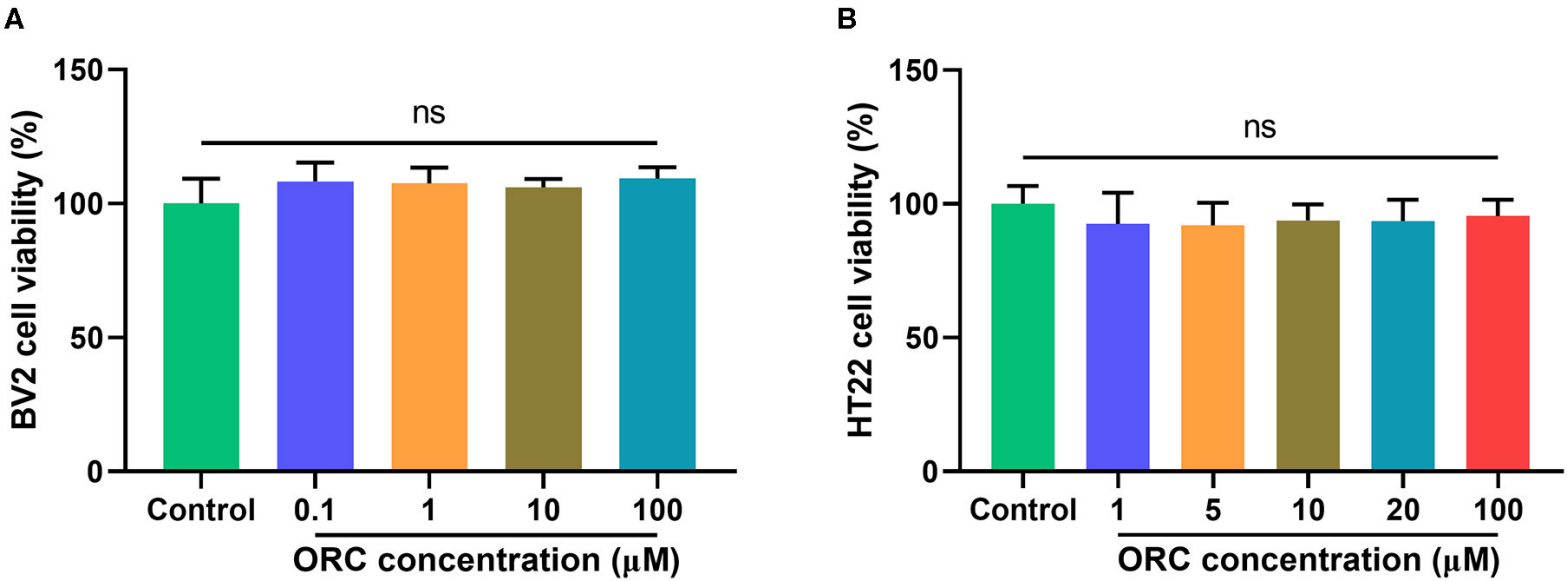
Cytotoxicity of oxiracetam (ORC) to BV2 microglial cells **(A)** and HT22 hippocampal cells **(B)**. The results are presented as the percentage of cell viability vs. controls, with control viability regarded as 100% (*n* = 6 for all groups). ns, not significant (*p* > 0.05).

The cytotoxicity of various concentrations of ORC on BV2 (0.1–100 μM) and HT22 (1–100 μM) cells was assessed using the MTT assay. Compared with controls, none of the concentrations of ORC exerted significant cytotoxic effects on BV2 and HT22 cells (*p* > 0.05 for all comparisons; Figures 1A,B). Therefore, the maximum concentration (100 μM of ORC) was used in further experiments.

### Oxiracetam Inhibits Aβ-Induced Morphological Changes and Increase in Phagocytosis in BV2 Cells

**Figure 2:**
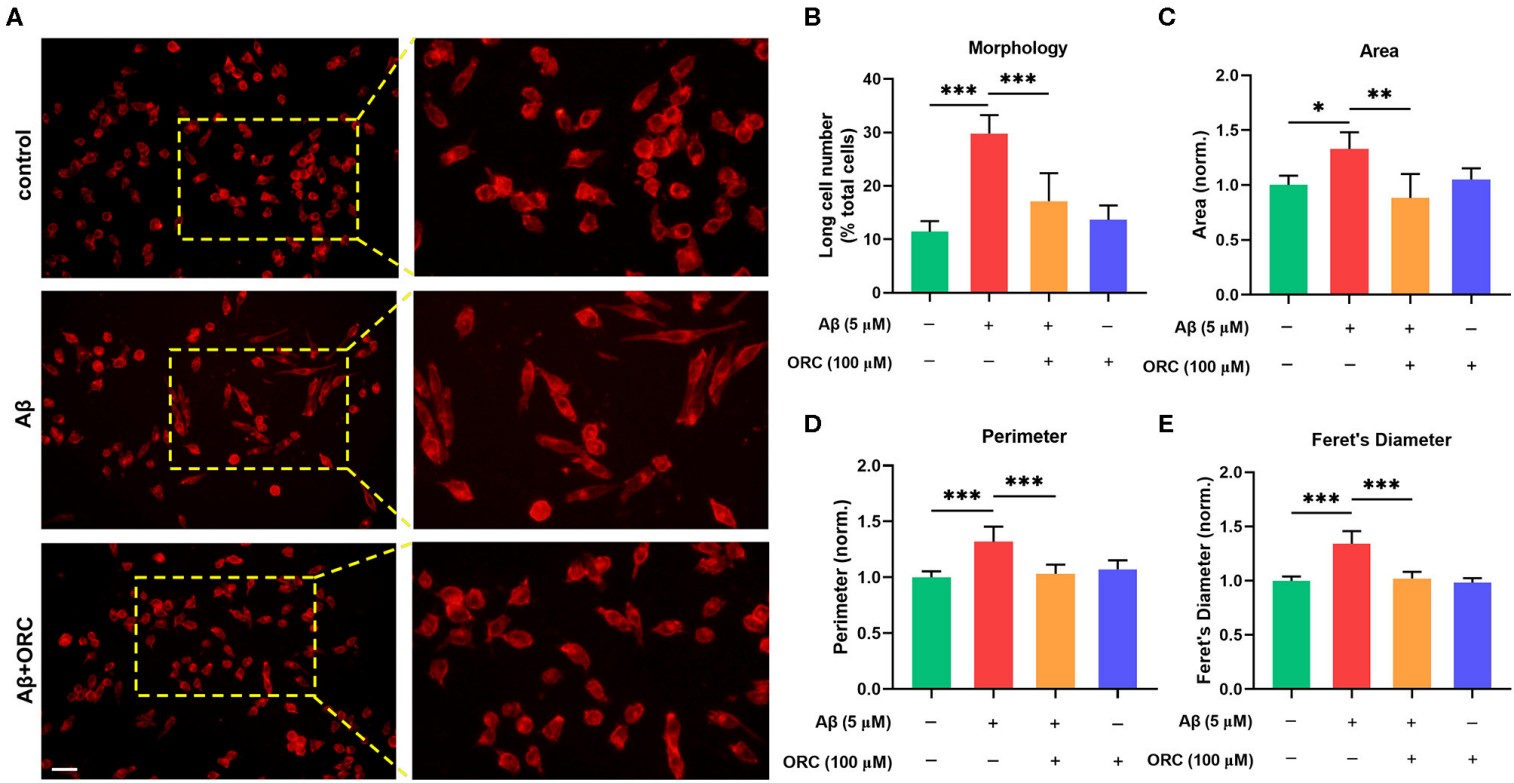
Oxiracetam (ORC) inhibits morphological changes induced by Aβ. The morphological changes in BV2 cells were assessed using ActinRed, and the “short” and “long” morphological phenotypes within BV2 cell populations were observed. Scale bar: 50 μm **(A)**. Stimulation with Aβ led to an increased ratio of cells with the “long” phenotype. ORC attenuated this change **(B)**. ORC modulated Aβ-induced BV2 cell morphological alterations in terms of cell area **(C)**, perimeter **(D)**, and Feret's diameter **(E)**. *p < 0.05, **p < 0.01, and ***p < 0.001.

**Figure 3:**
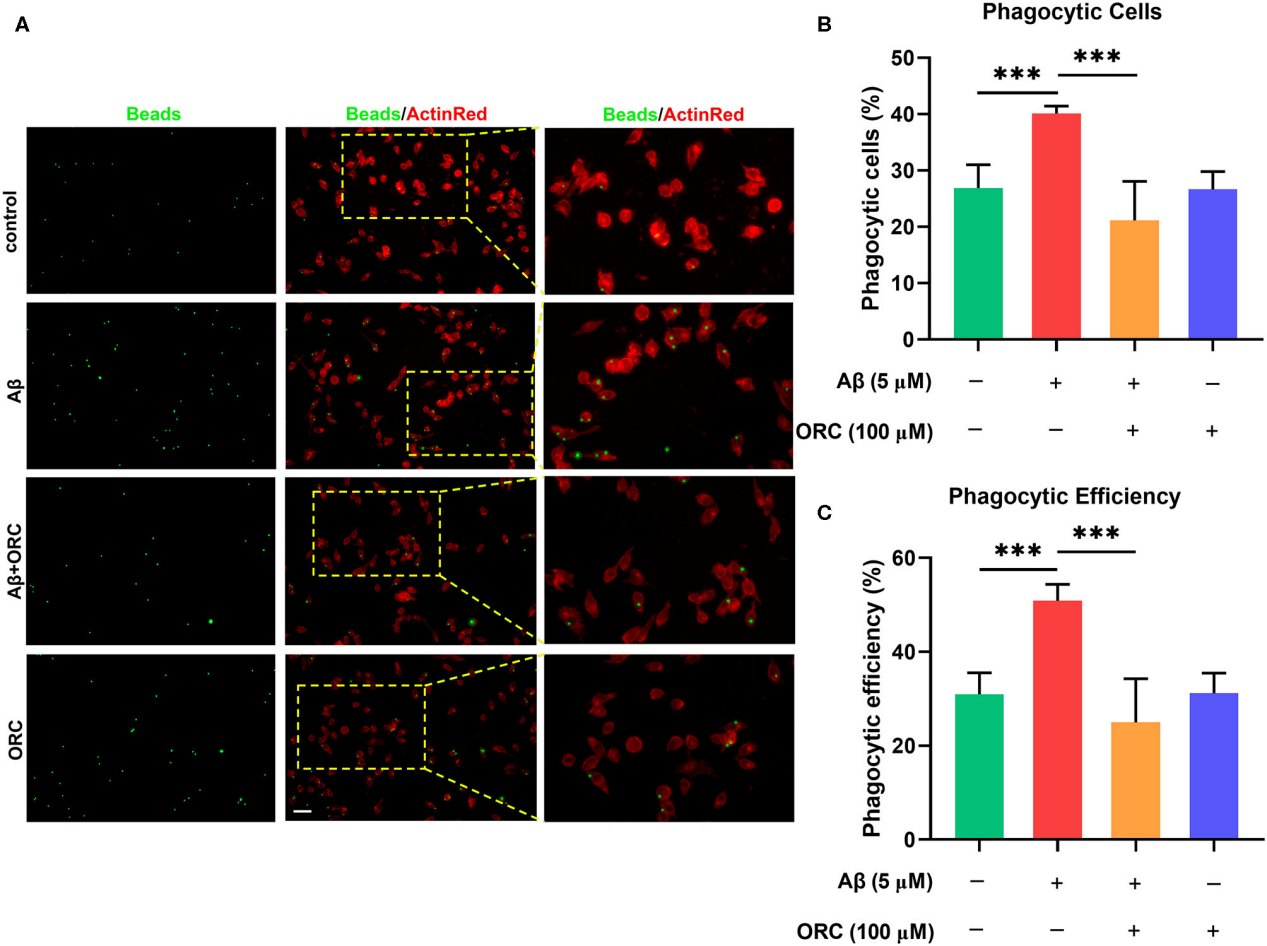
Inhibitory effects of oxiracetam (ORC) on Aβ-induced phagocytosis by BV2 microglial cells. **(A)** Representative immunofluorescence images of latex beads phagocytosed by ActinRed-marked BV2 cells. Scale bar: 50 μm. **(B)** Quantitative analysis of the percentage of phagocytic BV2 cells treated with Aβ and/or ORC. **(C)** Quantitative analysis of the phagocytic efficiency of BV2 cells based on a weighted average of ingested beads per cell. Data represent the mean ± standard deviation (*n* = 6 for all groups). ***p < 0.001.

On stimulation with Aβ, the morphology of BV2 cells changed from a short and compact state to an extended and elongated one (Figure 2A); however, ORC treatment reduced the ratio of elongated cells (Figure 2B). We further quantified three other parameters of BV2 morphology: cell area, perimeter, and Feret's diameter. As the results reveal, when BV2 cells were stimulated with Aβ, the cell area, perimeter, and Feret's diameter increased significantly. However, ORC treatment significantly reversed these morphological changes (Figures 2C–E). Given that activated microglia proliferate and concentrate around Aβ plaques and respond to neuroinflammation via phagocytosis, we further analyzed the morphological alterations of activated BV2 cells using the phagocytosis assay. To investigate the effect of ORC on the number of phagocytic cells and the weighted average of ingested beads per cell, which reflect the scale of BV2 cell activation and the phagocytic efficiency, respectively, a microglial phagocytosis assay was performed (Figure 3A). Fluorescence analysis shows that, compared with that of the control group, the percentage of phagocytic cells in the Aβ-treated BV2 cell group was increased by approximately 15% (26.90 ± 4.15% vs. 40.09 ± 1.34%, *p* < 0.001; Figure 3B). Moreover, ORC treatment significantly reduced the number of phagocytic cells by approximately 20% than did Aβ treatment alone (21.16 ± 6.88% vs. 40.09 ± 1.34%, *p* < 0.001; Figure 3B). We also analyzed the phagocytic efficiency of Aβ-treated BV2 cells with or without ORC treatment. Compared with Aβ treatment only, ORC treatment significantly reduced the phagocytic efficiency of BV2 cells by approximately 25% (50.89 ± 3.50% vs. 24.96 ± 9.30%, *p* < 0.001; Figure 3C). These results demonstrated that ORC can inhibit the activation and phagocytic ability of Aβ-treated BV2 cells.

### Oxiracetam Downregulates the Expression of Inflammatory Cytokines

**Figure 4:**
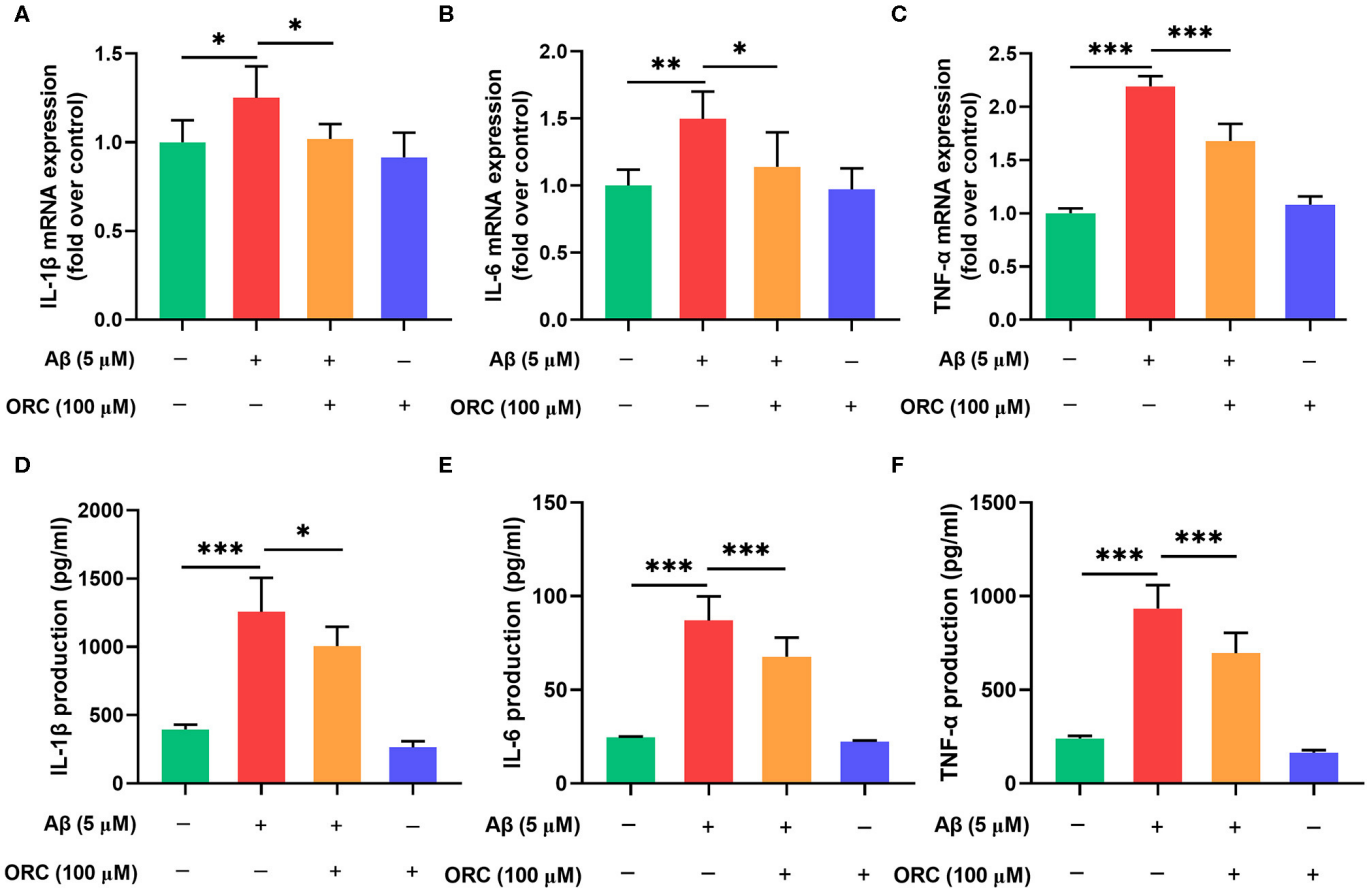
Inhibitory effects of oxiracetam (ORC) on pro-inflammatory cytokine mRNA and protein levels. BV2 microglial cells were treated with Aβ in the presence or absence of ORC. The mRNA levels of IL-1β **(A)**, IL-6 **(B)**, and TNF-α **(C)** were determined using qRT-PCR (*n* = 6 for all groups). The levels of IL-1β **(D)**, IL-6 **(E)**, and TNF-α **(F)** proteins were examined using ELISA (*n* = 7 for IL-1β and TNF-α, *n* = 9 for IL-6). All values are presented as the mean ± standard deviation. *p < 0.05, **p < 0.01, and ***p < 0.001.

It has been proved that Aβ oligomers stimulate the secretion of inflammatory molecules from microglial cells. Thus, to further investigate whether ORC has any inhibitory effects on the Aβ-induced increase in pro-inflammatory cytokine levels, we assessed the mRNA levels of IL-1β, IL-6, and TNF-α using qRT-PCR. The results showed that expression of IL-1β, IL-6, and TNF-α was significantly upregulated following treatment with Aβ compared with control. However, treatment with ORC downregulated the mRNA level of IL-1β (fold over control, Aβ = 1.25 ± 0.18; Aβ + ORC = 1.02 ± 0.08, *p* < 0.05; Figure 4A), IL-6 (fold over control, Aβ = 1.50 ± 0.20; Aβ + ORC = 1.14 ± 0.26, *p* < 0.05; Figure 4B), and TNF-α (fold over control, Aβ = 2.19 ± 0.10; Aβ + ORC = 1.68 ± 0.16, *p* < 0.001; Figure 4C). We also analyzed the mRNA level of IL-10, considered as a major anti-inflammatory cytokine, under Aβ stimulation, in the presence or absence of ORC. The results showed that Aβ did not significantly affect the mRNA level of IL-10 and that ORC did not affect the levels of this anti-inflammatory cytokine (fold over control, Aβ = 1.18 ± 0.2, *p* > 0.70 vs. control; Aβ + ORC = 0.98 ± 0.24, *p* > 0.60 vs. Aβ; Supplementary Figure 1).

In addition, we used ELISA to examine the expression of these proteins in the supernatant of BV2 cells stimulated with Aβ. Consistent with the results of the mRNA level analyses, the production of IL-1β (1,256.80 ± 248.48 vs. 395.28 ± 35.95 pg/ml, *p* < 0.001), IL-6 (87.11 ± 12.84 vs. 24.62 ± 0.50 pg/ml, *p* < 0.001), and TNF-α (933.80 ± 125.90 vs. 240.80 ± 14.01 pg/ml, *p* < 0.001) was increased upon Aβ induction than in the control group (Figures 4D–F). Pretreatment with ORC attenuated the production of IL-1β (*p* < 0.05), IL-6 (*p* < 0.001), and TNF-α (*p* < 0.001) by ~ 20, 23, and 25%, respectively, than did Aβ treatment only. These results show that ORC suppresses Aβ-triggered secretion of pro-inflammatory cytokines.

### Oxiracetam Inhibits Aβ-Induced Overproduction of Nitric Oxide

**Figure 5:**
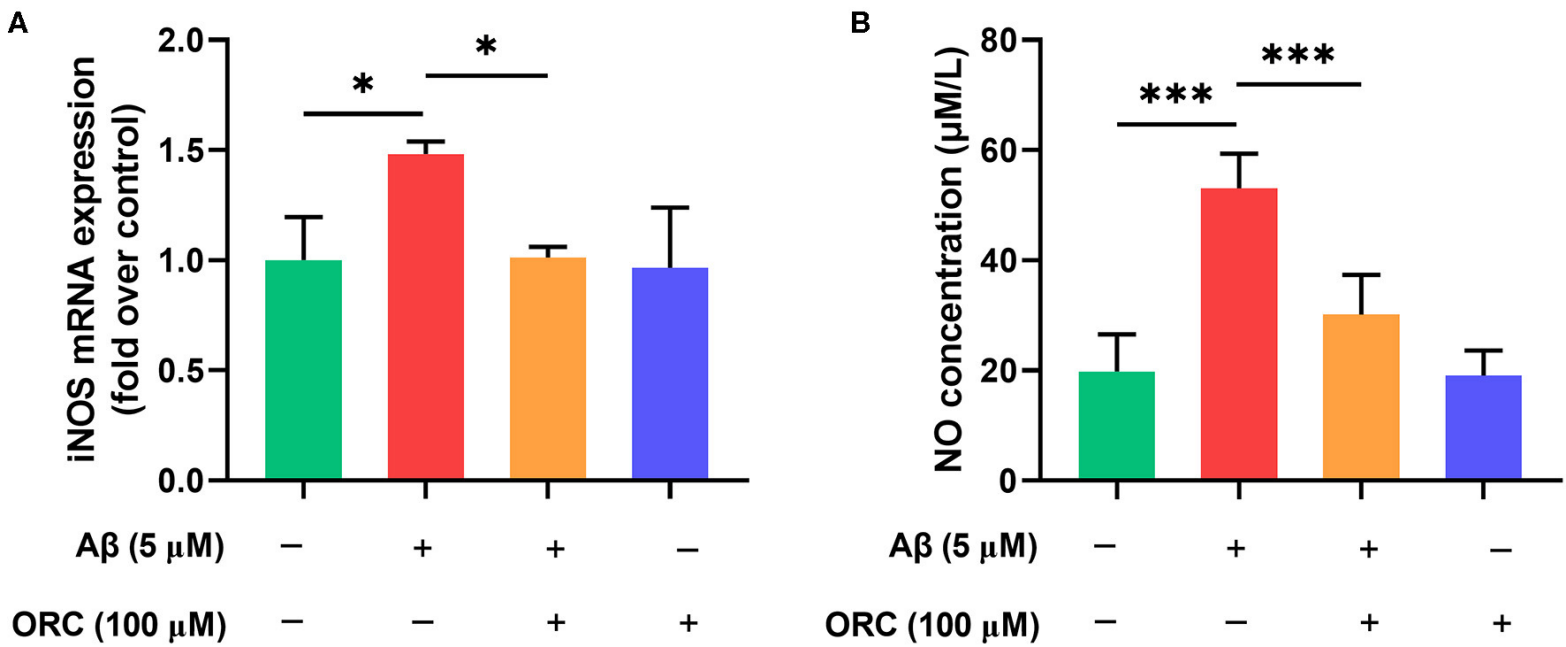
Inhibitory effects of oxiracetam (ORC) on the oxidative stress induced by Aβ in BV2 cells. BV2 microglial cells were treated with Aβ in the presence or absence of ORC. **(A)** The inducible nitric oxide synthase (iNOS) mRNA level was determined using qRT-PCR (*n* = 3 for all groups). **(B)** The level of nitric oxide (NO) in supernatants was examined using the NO assay (*n* = 5 for all groups). *p < 0.05 and ***p < 0.001.

Previous studies have demonstrated that Aβ can increase the levels of iNOS, which promotes the production of NO, in microglial cells. Oxidative stress is another damaging pathway that can lead to neuronal apoptosis via the overproduction of NO. Therefore, we next examined whether ORC has any effect on the levels of iNOS and NO. The results showed that level of iNOS mRNA was significantly upregulated upon stimulation with Aβ compared with that with control. However, treatment with ORC downregulated the iNOS mRNA level (fold over control, Aβ = 1.48 ± 0.06; Aβ + ORC = 1.01 ± 0.05, *p* < 0.05; Figure 5A). Furthermore, we showed that Aβ-stimulated BV2 cells significantly overproduced NO. However, ORC treatment reduced NO production significantly vs. Aβ alone (30.14 ± 7.2 vs. 53.09 ± 6.3 μM/L, *p* < 0.001; Figure 5B). Thus, ORC can inhibit Aβ-induced overproduction of NO in BV2 cells.

### Oxiracetam Protects HT22 Cells Against Aβ-Induced Neurotoxicity Indirectly

**Figure 6:**
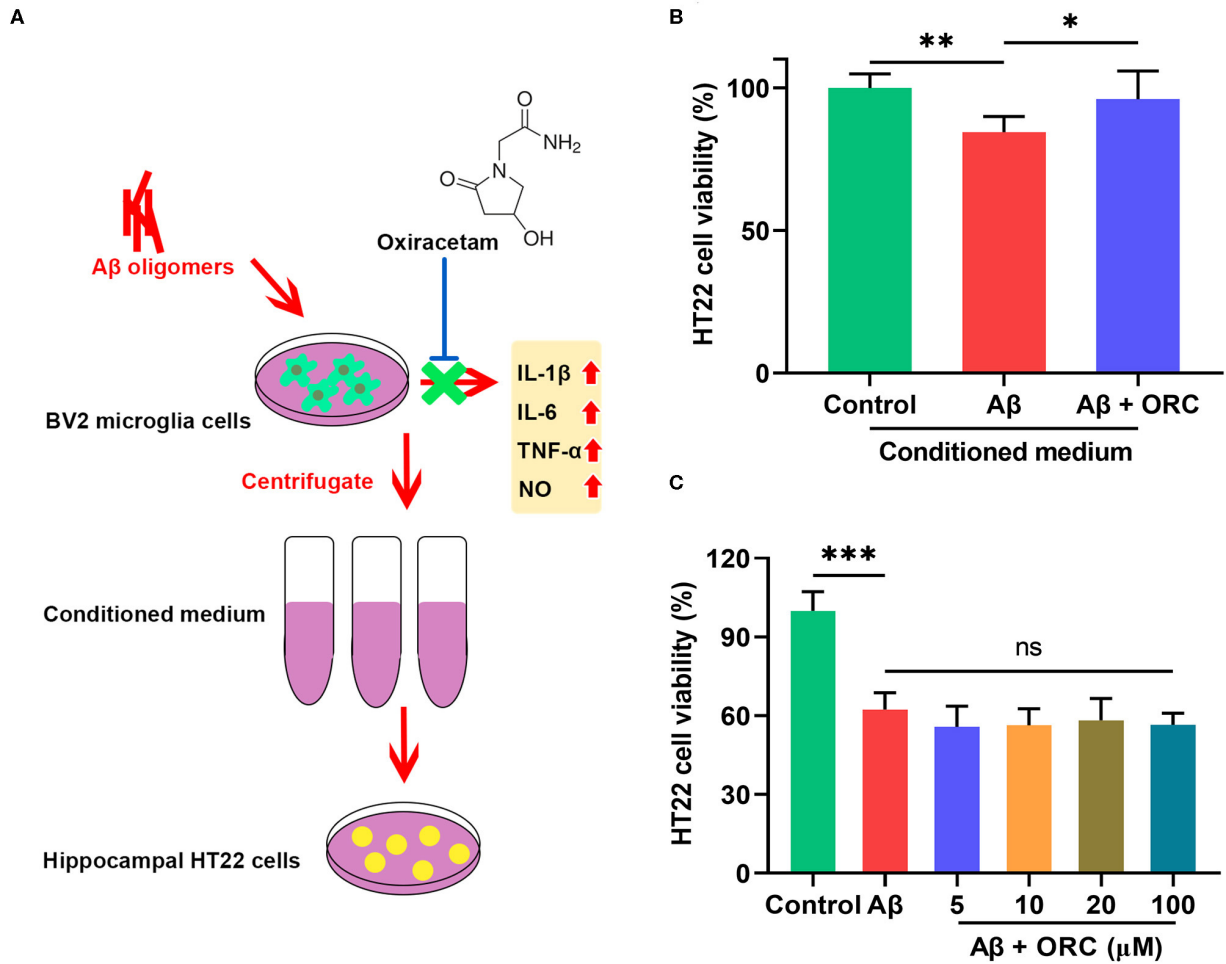
Neuroprotective effects of oxiracetam (ORC) against indirect toxicity of microglia-conditioned medium but not direct toxicity of Aβ. **(A)** Schematic of the experimental procedure to test the indirect toxicity of Aβ-induced BV2 cells in the presence or absence of ORC. **(B)** The results of the MTT assay revealed that ORC rescued the reduction of HT22 cell viability induced by conditioned medium from Aβ-induced BV2 cells. **(C)** The Aβ-induced decrease in HT22 cell viability was not affected by ORC (*n* = 8 for all groups). There was no significant difference between Aβ and Aβ + ORC groups. ns, not significant; *p < 0.05, **p < 0.01, and ***p < 0.001.

To determine whether the decreased production of pro-inflammatory cytokines and NO in Aβ-induced BV2 cells in the presence of ORC exerted a neuroprotective role, we next tested whether ORC could prevent the indirect toxicity of Aβ-stimulated BV2 cells on HT22 cells (Figure 6A). The viability of HT22 cells in the conditioned medium from Aβ-stimulated BV2 cells significantly decreased by almost 16% (vs. control, *p* < 0.01; Figure 6B). However, the viability of HT22 cells in conditioned medium from Aβ-induced BV2 cells co-cultured with ORC was increased (Aβ conditioned medium, 84.49 ± 5.44%; Aβ + ORC conditioned medium, 96.02 ± 9.86%, *p* < 0.05; Figure 6B). Thus, ORC protected HT22 cells against indirect Aβ-triggered toxicity. Next, we investigated whether ORC protected HT22 cells against direct Aβ toxicity. The results of the MTT assay revealed that treatment of HT22 cells with Aβ significantly reduced their viability (*p* < 0.001; Figure 6C). However, we did not find any direct protective effects ORC on Aβ-treated HT22 cell viability (*p* > 0.05; Figure 6C).

## Discussion

Accumulating clinical evidence indicates that ORC is beneficial for patients with cognitive impairment resulting from primary degenerative or multi-infarct dementia (20, 24). However, a previous study has shown that ORC does not significantly reduce cognitive impairment due to AD (25). Therefore, it is important to confirm whether ORC could play a protective role in AD. In our present study, we found that ORC inhibited Aβ-induced activation of microglia and attenuated the release of pro-inflammatory markers and NO. In addition, ORC protected hippocampal (HT22) against indirect toxicity from BV2 microglial cells. These findings are in line with the previously reported anti-inflammatory effects of ORC.

As the most important factor correlated with AD, activated microglia have been suggested to be related to amyloid plaque types and to contribute to neuroinflammation (26). Alterations in the phagocytic activity of microglia reflect the dynamic changes in microglial activation. During early AD pathogenesis, extracellular Aβ oligomers trigger a series of cascade reactions that lead to neuronal apoptosis and loss of neurons. Some studies have demonstrated that microglia can protect the brain by phagocytic clearance of damaged cells, debris, and Aβ aggregates, in part because this phagocytosis reduces inflammation (27). However, the deleterious role of phagocytosis in AD has also been reported. Recent studies investigated whether neuronal death mediated by Aβ-activated microglia resulted from the phagocytosis of viable neurons (28, 29). Activated microglia release TNF-α or other oxidants that cause the exposure of “eat-me” signals on the surface of live neurons, which evoked their phagocytosis by microglia (28, 30). In a mouse model of early phase AD, soluble Aβ oligomers induced the engulfment of synapses to contribute to cognitive decline in a CR3 pathway-dependent manner (12). Furthermore, microglia actively contribute to the amplification of tau aggregates via phagocytosis during AD pathogenesis (14). In the present study, we observed Aβ-induced BV2 phagocytosis of latex beads, which conceivably mimics the early microglial phagocytosis process; this process was significantly suppressed by ORC. To the best of our knowledge, no studies have explored the effects of ORC on the activation and phagocytosis of microglia in models of AD. Our results illustrate that ORC modulates Aβ-enhanced microglial phagocytosis. This modulation might be beneficial to the survival of neurons and synapses in AD.

As a response to Aβ stimulation, cultured microglial cells increase the expression of various cytokines, including IL-1β, IL-6, and TNF-α, as well as nitrogen species, some of which promote the sustained production of Aβ and subsequent continuous microglial activation in a vicious cycle that can cause deteriorative neuronal damage (31, 32). IL-1β, considered as a key modulator of the inflammatory response that can promote the release of other cytokines, including IL-6 (33), has been shown to exacerbate the typical pathologies of Aβ and tau accumulation (34, 35). In clinical practice, IL-6 has also been shown to be increased in the cerebrospinal fluid (36). A cohort study reported that individuals with elevated IL-6 level are at a greater risk of cognitive impairment (37). Anti-TNF-α drugs can reduce Aβ deposition and inflammation and rescue the behavioral performance in mouse models of AD, which suggests that TNF-α has an adverse effect on the progress of AD (38). Moreover, in response to Aβ, the overproduction of NO by activated microglia has also been verified in the progression of AD. NO can trigger inflammation and induce neuronal death via oxidative stress (39); therefore, reducing the levels of pro-inflammatory cytokines and NO released by activated microglia would be a promising strategy for the treatment of AD. In our study, ORC inhibited the elevation of IL-1β, IL-6, TNF-α, and NO levels in Aβ-stimulated BV2 cells; however, the levels were not restored to the baseline levels of control cultures. These results suggest that ORC may interfere with only a few inflammatory signaling pathways to block Aβ-induced increase in microglia-expressed cytokine levels. Other molecular mechanisms underlying the effects of ORC in AD models should be explored. For example, studies indicate that Aβ-induced prolonged activation of the toll-like receptor (TLR) pathway may be responsible for not only the aberrant phagocytosis process of microglia (28, 29, 40) but also the threatening oxidative stress and inflammatory responses (41). In addition, several studies have demonstrated the activation of PI3Kδ inhibited pro-inflammatory cytokine secretion through inhibition of TLR-mediated inflammation (42–45). A study has also demonstrated that S-ORC can reduce neuronal apoptosis by activating the PI3K signaling pathway (46). Collectively, we hypothesized that ORC may suppress Aβ-induced phagocytosis, microglial activation, and inflammation via modulating the TLR and/or PI3K signal pathway. We will verify this hypothesis in a further study.

Memantine and four cholinesterase inhibitors are the only approved symptomatic treatment drugs available for the treatment of AD. In particular, drugs meant to target Aβ pathology have faced setbacks again and again. This should serve as a reminder that the indirect toxicity caused by the Aβ cascade, involving phenomena such as inflammation and oxidative stress, deserved more attention. In the present study, ORC demonstrated the protective capacity to suppress the secondary damage derived from Aβ-induced activation of microglia, which indicates that ORC may also have a positive effect on indirect toxicity in complex *in vivo* systems. In addition, many previous studies have also reported that ORC improves abnormal mitochondrial oxidative phosphorylation and subsequent ATP metabolism (19, 47). ORC has also been shown to alleviate middle cerebral artery occlusion/reperfusion-induced BBB dysfunction, another pathology observed in patients with AD. So ORC may exert neuro-protection via multi-target strategies, such as improving energy metabolism, protecting the integrity of the BBB, and reducing inflammatory response, in AD. The mechanisms by which ORC confers neuroprotection should be further explored *in vivo* models of AD.

## Data Availability Statement

The original contributions presented in the study are included in the article/Supplementary Material, further inquiries can be directed to the corresponding author/s.

## Author Contributions

HZ performed experiments, analyzed data, and wrote the manuscript. LJ designed the study and reviewed the manuscript. JJ supervised the research, revised the manuscript, and obtained funding. All authors contributed to the article and approved the submitted version.

## Conflict of Interest

The authors declare that the research was conducted in the absence of any commercial or financial relationships that could be construed as a potential conflict of interest.
