# Supplementary material for: Oxiracetam Offers Neuroprotection by Reducing Amyloid β-Induced Microglial Activation and Inflammation in Alzheimer's Disease
Source: Front Neurol. 2020 Jul 17;11:623. doi: 10.3389/fneur.2020.00623 (PMC7380077; doi:10.3389/fneur.2020.00623)

**Supplementary Material**

**Supplementary Figure 1.** The effect of oxiracetam (ORC) on the mRNA level of interleukin-10 (IL-10). Aβ did not significantly affect the mRNA level of IL-10, and ORC did not affect the levels of this anti-inflammatory cytokine. (fold over control, Aβ = 1.18 ± 0.2, p > 0.70 vs. control; Aβ + ORC = 0.98 ± 0.24, p > 0.60 vs. Aβ) (n = 3 for all groups). ns, not significant (p > 0.05)


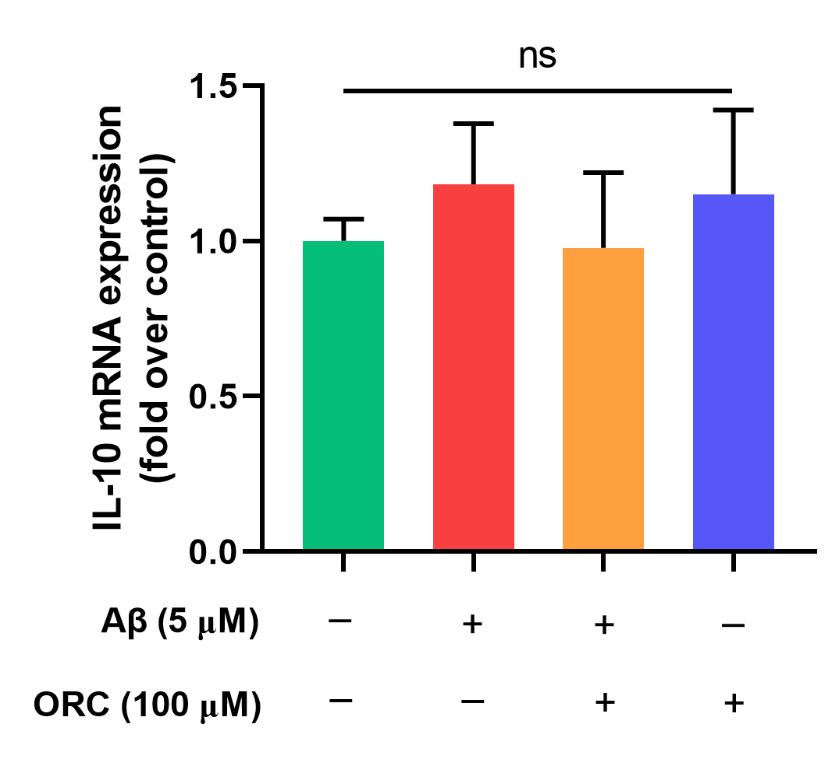

Supplement: Supplementary file 1 [file Data_Sheet_1.DOCX]
